# Editorial News and Miscellany

**Published:** 1886-10

**Authors:** 


### News and Miscellany.

The American Public Health Association will meet next year in Memphis.

Discontinued—From date, all clubbing arrangements are discontinued by mutual consent.

Dr. G. M. Boynton, late of Breckenridge, Stevens County, Texas, has settled in Austin. Welcome, Doctor.

The Mississippi Valley Medical Association will meet at Crab Orchard Springs, Kentucky, in July, next year. Let every State Association send delegates to swell the roll and push on the good work.

Physicians, Take Notice.—Physicians are requested to *pay no money* to any one traveling as agent, either for this Journal, or for the Physicians' Mutual Benefit Association of Texas. All authority to solicit for either is hereby revoked.

Dr. J. R. Briggs, our esteemed contemporary of the *Texas Courier Record of Medicine*, we regret to learn, has been seriously ill with phlegmonous erysipelas, and confined to bed at Bonham, Texas. The doctor had the misfortune to lose, by fire, at Bells, recently, his trunks, containing his wearing apparel, books, instruments, and valuable papers, to the amount of $400. Truly, misfortunes never come singly. The doctor has our sympathy and best wishes.

The Manufacture of Quinine by Synthesis.—Mr. Creswell Hewett, of London, has succeeded in manufacturing quinine synthetically from "an article which can always be got in abundance in any part of the world," and which is cheap. This discovery, it is thought, will reduce the price of quinine to a trifle (3d. per oz.) The importance of Mr. Hewitt's find will be appreciated by the British Government, which expends £60,000, or $300,000, annually in the cultivation of the Cinchona tree; and the saving will be understood, when it is remembered that the bark of the Cinchona only yields 2 per cent. of good quinine; 98 per cent. being valueless. Thus has CHEMISTRY contributed another great boon on mankind.

---

THE FEVER AT BOLOXI, MISS., AND "SHOTGUN" QUARANTINE.—After all the controversy about the nature of the fever which appeared at Boloxi, some weeks since, it seems to have turned out to be yellow fever, notwithstanding the possitive assertions to the contrary. Newspaper reports publish the number of cases at present at 275, and telegrams report the establishment of "shotgun quarantine" at neighboring towns.

It is too late in the season to fear any danger of its becoming epidemic in Texas; nevertheless Texas has re-established quarantine (on Oct. 19).

The sagacity of the Texas State Health Officer is apparent in his action in instituting quarantine when the fever was first reported, notwithstanding the diagnosis of "malaria," by the local doctors. It seems that Dr. Swearingen, who visited Boloxi, had his opinion of the fever, and acted on it.

---

TEXAS QUARANTINE OFFICERS AND THE STORMS.—If there are any who believe the position of Quarantine officer on the coast of Texas is a *sinecure*, let them ponder the recent experience of Drs. Fisher, Perkins and McFarland.

In the recent storm which swept away the beautiful city of Indianola, we learn that Dr. Fred K. Fisher, the quarantine officer at Pass Cavallo, with his wife, was only saved by clinging for nine long hours, waist deep, to some salt cedar bushes, while the waves broke often over their heads, and the current was so swift that it was with great difficulty they could keep their feet. The Doctor's house, with its entire contents, was carried away, including the old family silver, an heirloom in the family.

Dr. T. J. McFarland, at Indianola (who, however, is not a quarantine officer, but a practicing physician of that place, and an old resident), was driven, with his wife and children, to the house-top, whence they were washed off by the waves, and were saved only by clinging to floating timbers; while everything he possessed on earth, except his wife and children, was swallowed up and lost in the watery horror! Think of it! Imagine the situation, if you can—rendered doubly dreadful by the intense darkness of a storm-black night, whose gloom was intensified by the occasional lightning's flash, and the roar of the surging, angry sea!.

Dr. A. N. Perkins, the faithful quarantine officer at Sabine Pass, writes to State Health-Officer Swearingen, of the recent storm.

* * * "We lost everything but our lives. It is impossible to say now how many were lost; not less, I think, than seventy-five; only a small proportion of their bodies have been found. * Our town was almost entirely destroyed. The quarantine building has not yet been heard of. The life-saving building remains, not much injured. I will return to the Pass in a few days, and report to you, at an early date, what remains of the State property formerly in my charge."

In 1877, Dr. Pete, the Quarantine officer at Galveston, together with his grandson, and a fine saddle horse, were washed away, and we believe were never after heard of.

The above furnishes only another illustration of that self-sacrificing heroism so characteristic of the medical profession, which has prompted so many of its brilliant members to lay down their lives in the endeavor to discharge a sacred trust.

